# Inflammasome involvement in CS-induced damage in HaCaT keratinocytes

**DOI:** 10.1007/s11626-022-00658-x

**Published:** 2022-04-15

**Authors:** Roxane Prieux, Francesca Ferrara, Franco Cervellati, Anna Guiotto, Mascia Benedusi, Giuseppe Valacchi

**Affiliations:** 1 Department of Neurosciences and Rehabilitation, University of Ferrara, Ferrara, Italy; 2 Department of Environment and Prevention, University of Ferrara, Ferrara, Italy; 3 Plants for Human Health Institute, North Carolina State University, Kannapolis, NC USA; 4 Department of Food and Nutrition, Kyung Hee University, Seoul, 02447 South Korea

## Abstract

Cigarette smoke (CS) alters cutaneous biological processes such as redox homeostasis and inflammation response that might be involved in promoting skin inflammatory conditions. Exposure to CS has also been linked to a destabilization of the NLRP3 inflammasome in pollution target tissues such as the lung epithelium, resulting in a more vulnerable immunological response to several exogenous and endogenous stimuli related to oxidative stress. Thus, CS has an adverse effect on host defense, increasing the susceptibility to develop lung infections and pathologies. In the skin, another direct target of pollution, inflammasome disorders have been linked to an increasing number of diseases such as melanoma, psoriasis, vitiligo, atopic dermatitis, and acne, all conditions that have been connected directly or indirectly to pollution exposure. The inflammasome machinery is an important innate immune sensor in human keratinocytes. However, the role of CS in the NLRP1 and NLRP3 inflammasome in the cutaneous barrier has still not been investigated. In the present study, we were able to determine in keratinocytes exposed to CS an increased oxidative damage evaluated by 4-HNE protein adduct and carbonyl formation. Of note is that, while CS inhibited NLRP3 activation, it was able to activate NLRP1, leading to an increased secretion of the proinflammatory cytokines IL-1β and IL-18. This study highlights the importance of the inflammasome machinery in CS that more in general, in pollution, affects cutaneous tissues and the important cross-talk between different members of the NLRP inflammasome family.

## Introduction

Among the main sources of environmental pollutants, cigarette smoke (CS) is of important concern for its detrimental effects on human health, being considered the world’s leading preventable cause of death. The WHO declared that tobacco kills up around 8 million people, and the number of annual deaths is predicted to grow in the next few years (WHO 2000). Despite policy-based interventions such as tax increases on tobacco, health care prevention campaigns, and enforcing bans on advertising, cigarette consumption is still increasing in many countries and the epidemic is shifting towards the developing world (Schäfer *et al.* 2001; Sorg 2015). The CS aerosol is produced by incomplete combustion when burning a cigarette and can be divided into two phases: a particulate phase and a gas phase. The particulate phase constitutes 4–9% of the total smoke by weight, whereas the gas phase is the major fraction with 91–96% (Clunes *et al.* 2008). The most harmful CS components derived from the combustion of the cigarette and are part of the gas phase, which also included reactive aldehydes (formaldehyde, acrolein), reactive oxygen species (ROS), reactive nitrogen species (RNS), and hydrogen cyanide. The particulate phase includes polycyclic aromatic hydrocarbons (PAHs) and tobacco-specific nitrosamines (TSNAs). Beyond its well-known association with pulmonary and cardiovascular diseases, many evidence have also shown detrimental effects of CS on skin tissue (Boffetta *et al.* 1997; Puntoni *et al.* 2004; Wolf *et al.* 2012; Krutmann *et al.* 2017; Liu *et al.* 2018) that represents our first biological barrier with the outdoor chemical and physical stressors. More specifically, the oxidative compounds derived from incomplete combustion of the cigarette can affect the cutaneous tissue (Segre 2006). Inhaled side stream CS is approximately four times more toxic per gram of total particulate matter than mainstream CS (Farage *et al.* 2008).

CS has been linked to various dermatological conditions and pathologies: poor or delayed wound healing, squamous cell carcinoma, melanoma, acne, psoriasis, and eczema, but also premature skin aging (Morita 2007; Valacchi *et al.* 2012). As demonstrated in many studies, upon exposure to CS there is an increase of intracellular ROS that lead to an oxidative damage assessed in both in vitro and in vivo conditions (Bartecchi *et al.* 1995; Clunes *et al.* 2008; Jeong *et al.* 2010; Nakamura *et al.* 2013; Dong *et al.* 2016). This leads to the generation of reactive electrophilic molecules, such as reactive aldehydes (e.g., 4-hydroxy-2-nonenal (4-HNE)) and malondialdehyde (MDA) that can induce irreversible cell impairment (Dae *et al.* 2001; Nakamura *et al.* 2013; Cervellati *et al.* 2014; Chaichalotornkul *et al.* 2015). Indeed, due to the close interaction between lipids and proteins in tissues, these lipid peroxidation products can cause protein adduct formation and crosslinking, progressively leading to improper protein function and ultimately to cell dysfunction and apoptosis (Kennedy-Feitosa *et al.* 2016).

Besides affecting cellular redox homeostasis, CS components can provoke inflammatory skin responses. Numerous chemicals, especially PAHs, can penetrate the epidermal barrier and enter the systemic circulation through the capillaries present in the dermis causing systemic effects (Soeur *et al.* 2017). Inflammation is involved in most skin conditions including atopic dermatitis and psoriasis, and the consequent release of cytokines has been clearly shown to influence keratinocyte proliferation and differentiation (Bakhru and Erlinger 2005). At cellular level, in addition to the canonical inflammatory machinery activation, in 2002, Martinon and colleagues identified a new multiprotein signaling pathway, the inflammasome, involved in the activation of proinflammatory caspases (Martinon *et al.* 2002). The inflammasome activation seems to be correlated to numerous chronic and degenerative inflammatory diseases, such as type 2 diabetes, atherosclerosis, osteoarthritis (Grandemange *et al.* 2016; Sun *et al.* 2017; Azam *et al.* 2019), neurodegenerative diseases, Alzheimer’s disease (AD), Parkinson’s disease (PD), and cancer (Maryam Moossavi *et al.* 2020) but also to skin diseases (Fenini *et al.* 2020; Tang and Zhou 2020). The inflammasome is a multiprotein signaling platform that consists of nucleotide-binding domain leucine-rich repeat–containing proteins (NOD-like receptors, NLRs also known as NALPs), such as NLRP1 and NLRP3. Although there are nearby twenty mammalian NLR proteins, NLRP1 and NLRP3 have been well described in initiating inflammasome assembly and activation and seem to be the one more involved in skin responses (Schroder and Tschopp 2010). The identification by pattern recognition receptors (PRR) of danger-associated molecular patterns (DAMPs) and pathogen-associated molecular patterns (PAMPs) during infection and tissue damage enables NLRs–ASC (apoptosis-associated speck-like protein containing a CARD, containing a caspase-recruitment domain) interaction, inflammasome assembly, and finally its activation. Once the inflammasome assembles, ASC initiates the recruitment of pro-caspase-1 to the inflammasome complex and its activation. Active caspase-1 in turn cleaves the proinflammatory cytokines interleukin-1β (IL-1β) and interleukin-18 (IL-18) into their functional cytokine forms, which are then secreted and induce a tissue inflammatory status (Kelley *et al.* 2019). Among the different regulators of inflammasome, ROS seem to play a dual role as a trigger and effector in its activity (Abais *et al.* 2015). Of note, the ability to generate ROS and induce oxidative stress by CS has been well demonstrated in human tissues (Van Der Toorn *et al.* 2007; Faux *et al.* 2009; Gould *et al.* 2011). Concerning the involvement of inflammasome in skin homeostasis, it has been shown that both NLRP1 and NLRP3 are present within the cutaneous tissue and participate in various skin pathologies, such systemic lupus erythematosus, vitiligo, psoriasis, and atopic dermatitis (Tang and Zhou 2020). However, to the best of our knowledge, no previous research has focused on the effect of CS in inducing skin inflammasome assembly and activation.

Starting from these evidences, since both CS exposure and inflammasome activation are associated with the development/exacerbation of skin disorders, we assumed that in human keratinocytes CS exposure induces the assembly and activation of the cutaneous inflammasome through a redox-dependent mechanism. Once the exposure conditions have been validated, first we confirmed that CS exposure induced oxidative stress in human keratinocytes, in terms of lipid peroxidation and carbonyl formation. Next, we have demonstrated that CS exposure modifies the assembly of the cutaneous inflammasome, in terms of NLRP3/NLRP1 activation, ASC oligomerization, caspase-1 activation, and IL-1β and IL-18 expression level and secretion. With this knowledge, therapeutic solutions may be developed targeting the altered markers, hence counteracting the detrimental impact of CS or other outdoor oxidative stressors on human skin homeostasis.

## Materials and methods

### HaCaT cell culture and CS exposure

HaCaT cells, a spontaneously immortalized keratinocyte cell line, were grown in Dulbecco’s modified Eagle’s medium high glucose (Lonza, Milan, Italy), supplemented with 10% FBS, 100 U/mL penicillin, 100 µg/mL streptomycin, and 2 mM l-glutamine. Cells were tested for mycoplasms and for specific keratinocyte markers such as keratins. All cell cultures were performed at 37 °C in 5% CO_2_ and 95% air. Before the exposure, HaCaT cells were cultured into a 1% FBS-supplemented medium for overnight starvation to synchronize to the same cell cycle phase. They were then exposed to either one 3R4F research cigarette or ambient air for different exposure times (ranging from 5 to 30 min) by using a vacuum pump able to burn the research cigarette, as previously described (Muresan *et al.* 2018; Prieux *et al.* 2020). At the end of the exposure, fresh media supplemented with 10% FBS were added to the cells and then, they were incubated for 24 h at 37 °C, 98% humidity, and 5% CO_2_. For the determination of CS exposure conditions, negative controls consisting non-exposed cells (air) were left incubated at 37 °C, 98% humidity, and 5% CO_2_ during the exposure time.

### MTT viability assay

HaCaT viability test upon CS exposure was assessed by the MTT assay (3-(4,5-dimethylthiazol-2-yl)-2,5-diphenyl tetrazolium bromide) which consists the mitochondrial-dependent reduction of 3-(4,5-dimethylthiazol-2-yl)-2,5-diphenyltetrazolium bromide (MTT) to formazan (Cavicchio *et al.* 2017). Briefly, HaCaT cells were seeded in 96-well plates at a density of 2 × 10^4^ cells/well in 200 μL of media and then exposed to CS for 5, 10, 15, and 30 min. Right after the end of exposure, cells were incubated for 24 h with fresh media. After complete removal of the media to avoid any color interference, 50 μL of serum-free media and 50 μL MTT (0.5 mg/mL) were added and incubated for 3 h. A total of 100 μL of DMSO was then added to dissolve the insoluble purple formazan crystals at 37 °C for 15 min. After shaking, the solution absorbance was measured at *λ* = 590 nm and subtracted from *λ* = 620 nm. LPS (10 and 100 μg/mL) was used as positive control.

### Immunocytochemistry

HaCaT cells were grown on coverslips in a 12-well plate at a density of 1 × 10^5^ cells/mL as previously described (Pambianchi *et al.* 2020). After CS/air exposure, cells were fixed in 4% paraformaldehyde (PFA) in PBS for 10 min at RT. Cells were then permeabilized for 10 min with 0.4% of Triton X-100 in PBS and then blocked in PBS containing 1% BSA at RT for 1 h. Coverslips were then overnight incubated at 4 °C with primary antibodies diluted in 0.25% BSA in PBS-T:4-HNE (AB5605, Merck, Darmstadt, DE) 1:200, NF-κB p65 (NB100-56712SS, Bio-Techne, Minneapolis, MN) 1:400, ASC (cat. NBP1-78,977, NovusBio, Centennial, CO) 1:100, and NALP1 (B-2) (cat. sc-166368, Santa Cruz, Dallas, TX) 1:50. The following day, coverslips were incubated at RT for 1 h with fluorochrome-conjugated secondary antibodies goat anti-mouse IgG (ab150116 Alexa Fluor 594, Abcam, Cambridge, MA) and goat anti-rabbit IgG (ab150077 Alexa Fluor 488, Abcam). After two 10-min washes with PBS-T, nuclei were stained with a 300 mM DAPI solution (Tocris Biosciences, Bristol, UK) for 5 min followed by three washes of 5 min with PBS-T. Coverslips were mounted onto glass slides using PermaFluor® Aqueous Mounting Medium (TA-006-FM, Thermo Fisher Scientific, Waltham, MA), and examined using a fluorescent microscope (Nikon Microphot FXA microscope; Nikon Instruments, Amsterdam, NL), and fluorescence intensity was quantified using ImageJ.

### Protein carbonyls (OxyBlot assay)

OxyBlot™ Protein Oxidation Detection Kit (Millipore, Burlington, MA) was used as a methodology for the detection and quantification of proteins modified by oxygen free radicals and other reactive species (Valacchi *et al.* 2015). A total of 20 μg of proteins extracted in a lysis buffer containing 1% 2-mercaptoethanol as a reducing agent was subjected to a derivatization reaction; i.e., the carbonyl groups in the protein side chains are derivatized to 2,4-dinitrophenylhydrazone (DNP-hydrazone) by reaction with 2,4-dinitrophenylhydrazine (DNPH). Proteins which have undergone oxidative modification will be identified by appearing as a band only in the lane containing the derivatized sample, but not in the lane containing the non-derivatized proteins, used as negative controls. As an internal control, a standard mixture composed of five proteins containing 1–3 DNP residues was used. This standard serves as an internal control for the electrophoresis, Western transfer, and immunodetection steps of the OxyBlot™ procedure. The DNP-derivatized and non-derivatized protein samples are separated by a 12% polyacrylamide gel, electroblotted, and blocked for 1 h in PBS 0.5% Tween 20, containing 5% milk. Membranes were incubated with primary antibody, specific to the DNP moiety of the proteins. This step was followed by incubation with a horseradish peroxidase-antibody conjugate directed against the primary antibody (secondary antibody: goat anti-rabbit IgG). The filters were then treated with chemiluminescent reagents (luminol and enhancer). The luminol is converted to a light-emitting form at wavelength 428 nm by the antigen/primary antibody/secondary antibody/peroxidase complex (bound to the membrane) in an H_2_O_2_ catalyzed oxidation reaction.

### Protein extraction and Western blotting

Cell lysates were washed in PBS, centrifuged, and then extracted in ice-cold RIPA buffer 1X containing 50 mM Tris (pH 7.5), 150 mM NaCl, 10% glycerol, 1% Nonidet P-40, 1 mM EDTA, 0.1% SDS, 5 mM N-ethylmaleimide, and 10% protease inhibitor cocktail (100 μL for a total volume of 1 mL) (SIGMAFAST™ Protease Inhibitor Tablets, Merck), β-glycerol phosphate (10 μL for a total volume of 1 mL), and orthovanadate (5 μL for a total volume of 1 mL) (Valacchi *et al.* 2009). Lysates were cleared by centrifugation (13,000 rpm) for 15 min at 4 °C, and protein concentration was quantified using the Bradford protein assay with absorbance measurement at 595 nm (Bio-Rad, Protein Assay; Bio-Rad Laboratories, Inc., Milan, Italy). Equivalent amounts of proteins (40 μg) were loaded onto 6–15% polyacrylamide SDS gels and separated by molecular size. Gels were electroblotted onto nitrocellulose membranes and then were blocked for 1 h in PBS, pH 7.5, containing 0.5% Tween 20 and 5% milk. Membranes were incubated overnight at 4 °C under gentle rocking with primary antibodies diluted in 1% non-fat milk PBS-T. Primary antibodies used include ASC (cat. NBP1-78,977, NovusBio) 1:1000, caspase-1 (14F468) (cat. sc-56036, Santa Cruz) 1:100, and NALP3 (NBP2-12,666, NovusBio) 1:1000. The membranes were then incubated with horseradish peroxidase conjugated secondary antibodies for 2 h; anti-rabbit (cat. AB6721, Abcam) and anti-mouse (cat. BAF007, Bio-Techne) and the bound antibodies were detected by chemiluminescence using the ECL kit reagents WESTAR ETAC ULTRA 2.0 (cat. XLS075, 0100, CYANAGEN, Bologna, Italy) and Bio-Rad ChemiDoc™ imaging system. β-Actin (cat. A3854, Merck) was used as loading control. Images of the bands were digitized, and densitometry analysis was performed using ImageJ software.

### Nuclear-cytosolic protein extraction

HaCaT cells were seeded in 100-mm Petri dishes (3 × 10^6^ cells) and allowed to grow at the confluence. After 15 min of CS/air exposure, cells were detached at different time points, washed with ice-cold PBS 1X, and pelleted by centrifugation. Cell pellets were processed as previously described. Briefly, cell pellets were resuspended in the cytosolic extraction buffer containing 10 mmol/l HEPES (pH 7.9), 10 mmol/l KCl, 1.5 mmol/l MgCl_2_, 0.3% Nonidet P-40, 0.5 mmol/l dithiothreitol, 0.5 mmol/l phenyl-methylsulphonyl fluoride, and protease and phosphatase inhibitor cocktails. The lysates were incubated for 15 min on ice and further destructed by using a G27 gauge syringe. Cell suspensions were centrifuged for 15 min at 10,000 rpm, 4 °C. The supernatants containing the cytoplasmic fraction were collected whereas the remained pellets containing the nuclei were suspended in the nuclear extraction buffer containing 20 mmol/L HEPES (pH 7.9), 0.6 mol/L KCl, 1.5 mmol/L MgCl_2_, 20% glycerol, 0.5 mmol/L phenyl-methylsulphonyl fluoride, and protease and phosphatase inhibitor cocktails and destructed using a G27 gauge syringe. Samples were then incubated in ice with intermitted mixing for 60 min and then centrifuged at 12,400 rpm for 5 min at 4 °C to obtain the nuclear protein fractions. Protein concentration was determined using a Bradford protein assay (Bio-Rad Protein Assay, Bio-Rad Laboratories Inc.). A total of 30 μg of proteins was then loaded onto 10% polyacrylamide SDS gels and separated by molecular size. Gels were electroblotted onto nitrocellulose membranes and then were blocked for 1 h in PBS, pH 7.5, containing 0.5% Tween 20 and 5% milk. Membranes were incubated overnight at 4 °C under gentle rocking with primary antibody NF-κB p65 D14E12 (cell signaling, cat. 8242) diluted 1:1000 in 1% non-fat milk in PBS-T. α-Tubulin (B-5–1-2) (sc-23948, Santa Cruz Biotechnology, Inc.) and lamin A/C (E-1) (sc-376248, Santa Cruz Biotechnology, Inc.) were used as loading controls at a concentration of 1:500 in PBS-T, 1% non-fat milk. The membranes were then incubated with horseradish peroxidase conjugated secondary antibodies anti-rabbit (ab6721, Abcam) or anti-mouse (DkxMu-003-D, Immuoreagents Inc., Burlington, NC) for 90 min at RT. The bound antibodies were detected by chemiluminescence using the ECL kit reagents WESTAR ETAC ULTRA 2.0 (CYANAGEN) and Bio-Rad ChemiDoc™ imaging system. Images of the bands were digitized, and densitometry analysis was performed using ImageJ software.

### ASC oligomerization

HaCaT cells were grown in 60-mm Petri dishes at a density of 1.5 × 10^6^ cells in 3 mL of media. After 24 h, cells were exposed to CS/air for 15 min and then collected 6 h (T6) after CS exposure. Samples were used for oligomerization on the same day of collection. Cell samples were washed in cold PBS and centrifuged for 5 min at 1500 × *g* at 4 °C. The resulting cell pellet was resuspended in 200 μL of ice-cold lysis buffer containing HEPES KOH 20 mM (pH 7.5), KCl 150 mM, 1% NP-40, 1% protease inhibitor cocktails (Merck), and 0.1 mM PMSF protease inhibitor (cat. 36,978, Thermo Fisher Scientific). The cell lysates were left on ice for 10–15 min, vortexed every 5 min, and then centrifuged for 8 min at 1800 × *g* at 4 °C to remove bulk nuclei. A volume of 30 μL of the lysates was collected as input for Western blot analysis. The remaining volume of the cell lysates was then centrifuged for 10 min at 5000 × *g* at 4 °C, resuspended in 0.5 mL of cold PBS containing 2 mM of disuccinimidyl suberate, DSS (CAS 68528–80-3 Alfa Aesar, Thermo Fisher Scientific) for crosslinking oligomers, and incubated at RT for 30 min on a rotator. After 30 min, the samples were centrifuged for 10 min at 10,000 × *g* at 4 °C, and the crosslinked pellets were then resuspended in 1 × Laemmli buffer. The input and crosslinked samples were boiled for 10 min at 95 °C and then analyzed by running samples on a 12% SDS-PAGE gel.

### RNA extraction and quantitative RT-PCR (qRT-PCR)

For RNA extraction and consequent qRT-PCR, HaCaT cells were seeded in 6-well plates at a density of 0.9 × 10^6^ cells and then exposed for 15 min to CS. Total RNA was extracted following the phenol–chloroform extraction protocol described by Frigato *et al.* (2020) adopting some modifications. Briefly, after washing HaCaT cells twice in PBS, 500 μL of Pure-ZOLTM RNA Isolation Reagent (Bio-Rad, Milan, Italy) was added. Cell suspension was collected in 1.5-mL Eppendorf tubes and then, 100 μL of chloroform was added to separate the different organic phases by centrifuging the cell suspension at 12,000 rpm for 15 min at 4 °C. The upper aqueous phase containing RNA was collected in new Eppendorf tubes and the chloroform step was repeated. To precipitate the RNA pellet from the aqueous phase, 250 μL of methanol was added and samples were centrifuged at 12,000 rpm for 10 min at 4 °C. The RNA pellet was washed 3 times in 1 mL of 75% ethanol and centrifuged at 12,000 rpm for 10 min at 4 °C. After left the remaining ethanol evaporate under the chemical hood for 3–5 min, RNA pellet was suspended in 25 μL of nuclease-free water. RNA concentration was measured using the Shimadzu BioSpec-nano spectrophotometer (Shimadzu Biotech, Duisburg, DE). Next, cDNA was generated from 1 μg of total RNA, using the iScript cDNA Synthesis Kit (Bio-Rad). The mRNA levels of *IL-1β* and *IL-18* were evaluated by quantitative real-time PCR using the SYBR® Green Master Mix (Bio-Rad) on a CFX Connect Real-Time PCR System (Bio-Rad) following the manufacturer’s protocol. Gene expression was quantified by obtaining the number of cycles to reach a predetermined threshold value in the intensity of the PCR signal (Ct value). *RPL11* was used as reference gene and samples were compared using the relative cycle threshold (Ct). After normalization, quantitative relative gene expression was calculated by the 2^−∆∆Ct^ method.

### Detection of IL-1β and IL-18 using ELISA assays

IL-1β and IL-18 levels were measured in media of HaCaT cells harvested 24 h upon CS exposure using the Human IL-1β/IL-1F2 DuoSet ELISA (cat DY201-05 R&D System, Bio-Techne) and the Human IL-18/IL-18 BPa Complex DuoSet ELISA (cat. DY8936-05 R&D System, Bio-Techne) according to the manufacturer’s instructions. IL-1β/ IL-18 levels were expressed as pg/mL. Gen5 software (Agilent, BioTek, Santa Clara, CA) was used for the detection.

### Statistical analysis

For each experiment, analysis of variance (one-way ANOVA), followed by the Bonferroni post hoc test, was used. Statistical significance was considered at *p* < 0.05. Data are expressed as mean ± SD of duplicate determinations obtained in at least 3 independent experiments.

## Results

### Cigarette smoke affects human keratinocyte redox homeostasis

**Figure 1.**
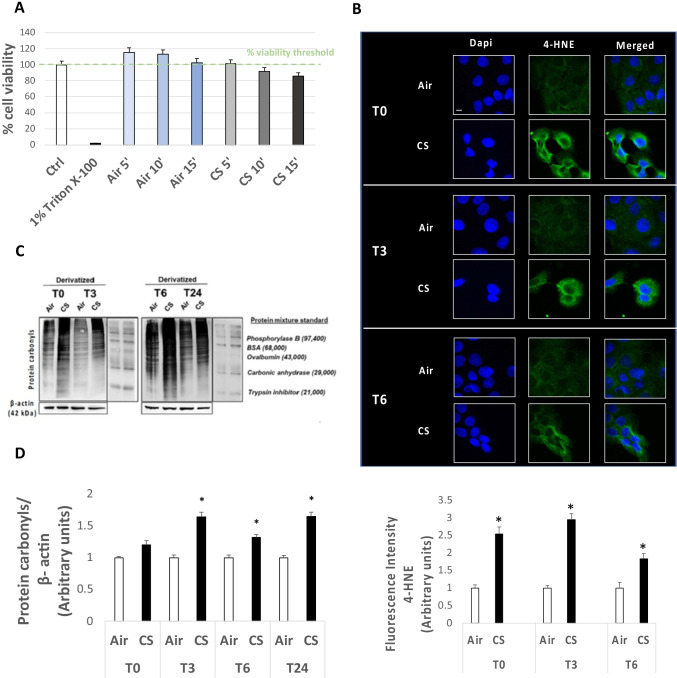
(*A*) Cell viability evaluated by MTT assay on HaCaT cells after exposure to CS for 5, 10, and 15 min compared to control (ctrl) samples (air); 1% Triton X-100 was used as positive control. (*B*) Immunocytochemical staining for 4-HNE protein adducts in HaCaT cells 0, 3, and 6 h (T0, T3, and T6) after 15 min of CS exposure or unexposed (air; green staining represents 4-HNE, and the blue staining (DAPI) represents nuclei). Images were taken at 40 × magnification; *scale bar* = 20 μm. The* bottom panel* displays the quantification of immunofluorescence staining intensity for 4-HNE. (*C*) Western blotting of protein carbonyl levels of HaCaT cells exposed for 15 min to CS and harvested right after the end of exposure (T0), and 3, 6, and 24 h after the 15-min exposure (T3, T6, and T24), by means of the OxyBlot assay. β-Actin was measured on non-derivatized samples as a normalization protein. The protein mixture standard is DNP-bovine serum albumin (BSA) used as internal control provided by the OxyBlot detection kit (see “Materials and methods”). (*D*) Quantification of protein carbonyl level of CS-exposed HaCaT cells respect to the control (air) at the different time points (T0, T3, T6, and T24), by using the ImageJ software. Data are the results of the averages of at least three different experiments, *p < 0.05 by one-way ANOVA

Preliminary experiments were performed to evaluate the highest nontoxic dose of CS to be used in our experimental procedure. As depicted in Fig. 1*A*, 15 min with one cigarette resulted to be the best approach as 24 h after exposure cell viability was over 85%. To evaluate whether the selected CS condition was sufficient to increase oxidative stress markers, 4-hydroxy-nonenal (4-HNE) levels were assessed by immunofluorescence staining. As shown in Fig. 1*B*, HaCaT cells exhibited an increased signal for 4-HNE staining immediately after CS exposure, when compared to the air-treated cells. This trend was maintained also at T3, with a small decrease 6 h (T6) after CS exposure (Fig. 1*B*).

In addition to 4HNE, which is a marker of peroxidation, we have confirmed the increased oxidative stress level also by measuring proteins carbonylation formation, which is considered another hallmark of redox damage. As it is shown in Fig. 1*C*, *D*, there was a significant increase in protein carbonyl level in cells exposed to CS after 3, 6, and 24 h when compared to unexposed cells. Taken together, the results confirmed that the selected CS exposure conditions were able to induce an oxidative damage in HaCaT cells without affecting cell viability.

### CS exposure inhibits NLRP3 inflammasome activation in HaCaT cells

**Figure 2.**
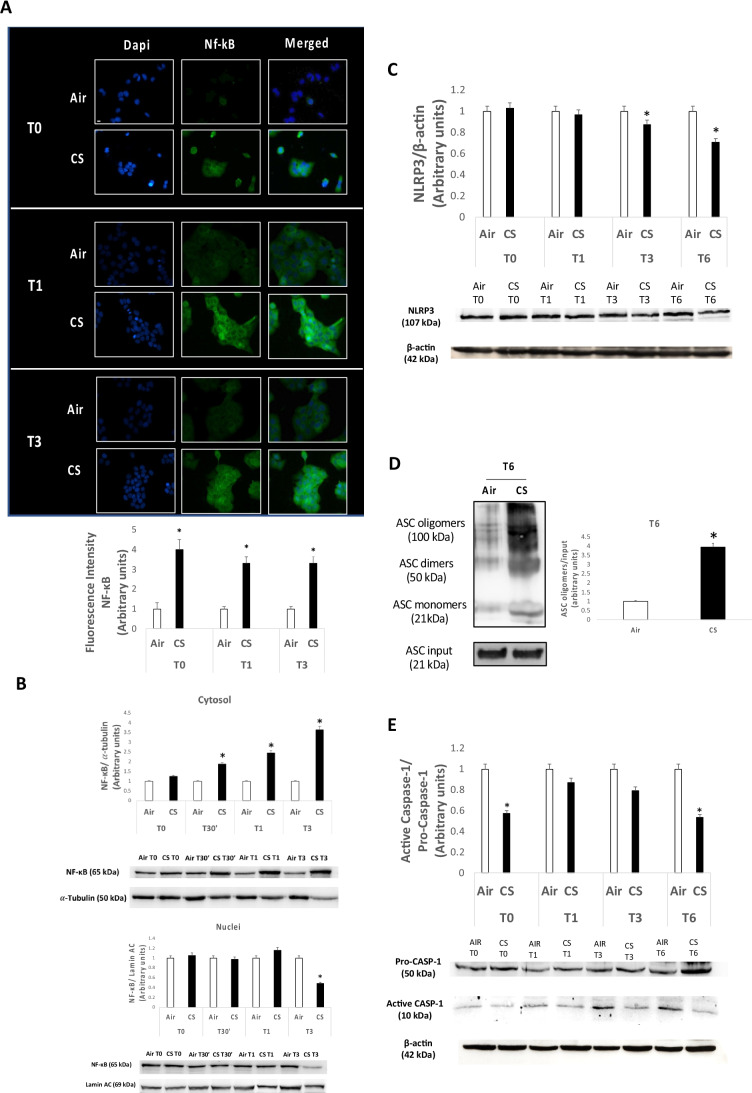
(*A*) Immunocytochemical analysis of NF-κB in HaCaT cells exposed to CS for 15 min compared to ctrl samples (air), 0, 1, and 3 h (T0, T1, and T3) upon the end of 15-min CS exposure. *Green staining* represents NF-κB, while DAPI fluorescent stain (*blue staining*) was used to counterstain the cell nuclei. Pictures were taken at 40 × magnification; *scale bar* = 20 μm. Fluorescence intensity for NF-κB was measured using ImageJ software. (*B*) Protein expression levels of NF-κB in cytosol (*upper panel*) and nuclei (*below panel*) in HaCaT cells exposed to CS for 15 min and collected at the selected time points starting from the end of the CS insult, with relative expression level quantification. α-Tubulin and lamin A/C were used as loading control for cytoplasm and nuclei fractions respectively. ImageJ software was used for the analysis. (*C*) Protein expression levels of NLRP3 in HaCaT samples exposed to CS and collected 0, 1, 3, and 6 h post 15 min of CS exposure. The NLRP3 expression level was quantified using ImageJ software and β-actin was used as internal control. (*D*) ASC oligomer levels in HaCaT samples 6 h (T6) post 15-min CS exposure with relative expression level quantification. β-Actin was used as internal control. (*E*) Protein expression levels of caspase-1 0, 1, 3, and 6 h (T0, T1, T3, and T6) after 15 min of CS exposure in HaCaT cells. The *upper panel* displays the quantification of the ratio between active caspase-1 and pro-caspase-1; ImageJ software was used for the analysis. Data are the results of the averages of at least three different experiments, *p < 0.05 by one-way ANOVA

The nuclear factor kappa-light-chain-enhancer of activated B cells (NF-κB) is one of the main redox-sensitive transcription factors involved in the inflammatory response of our body. CS is known to modulate NF-κB pathway, by altering the cellular redox homeostasis (Anto *et al.* 2002; Liu *et al.* 2008). Indeed, the cross-talk between oxidative and inflammatory pathways (OxInflammation) is now a well-known event occurring in a variety of conditions including cutaneous tissue exposed to air pollutant stimuli (Valacchi *et al.* 2018; Ferrara *et al.* 2020). Since we found that CS was able to induce an oxidative damage in human keratinocytes, we wanted to investigate whether also NF-κB might be affected by the CS exposure. As it is shown by immunofluorescence (Fig. 2*A*), keratinocytes exposed to CS expressed high levels of cytoplasmic NF-κB subunit at 0, 1, and 3 h (T0, T1, and T3) with respect to the unexposed cells. However, the increased levels of total NF-κB were not followed by an increased nuclear translocation in CS-exposed cells. These data were also confirmed by Western blot analysis (Fig. 2*B*), with a time-dependent increase of NF-κB expression level within cytosol not followed by a nuclear translocation of NF-κB in nucleus. Considering that increased NF-κB nuclear translocation is a crucial step in NLRP3 inflammasome activation and expression, we have analyzed NLRP3 levels under our experimental conditions. We found that CS exposure induced a decrease in the protein expression levels of NLRP3 6 h after CS exposure (Fig. 2*C*). Interestingly, higher oligomer expression levels of the speck-like receptor ASC, which are generated upon NLRP3 activation to form the active multiprotein inflammasome scaffold, were revealed 6 h after CS exposure (Fig. 2*D*). However, despite the increased ASC oligomer levels, CS exposure negatively modulated the activation of the ICE-converting enzyme caspase-1, usually activated upon inflammasome scaffold formation and responsible of the maturation of interleukins IL-1β/IL-18 (Fig. 2*E*). These data suggest the ability of CS to downregulate NLRP3 inflammasome expression and activation.

### Inflammasome NLRP1 is activated in HaCaT cells exposed to CS

**Figure 3.**
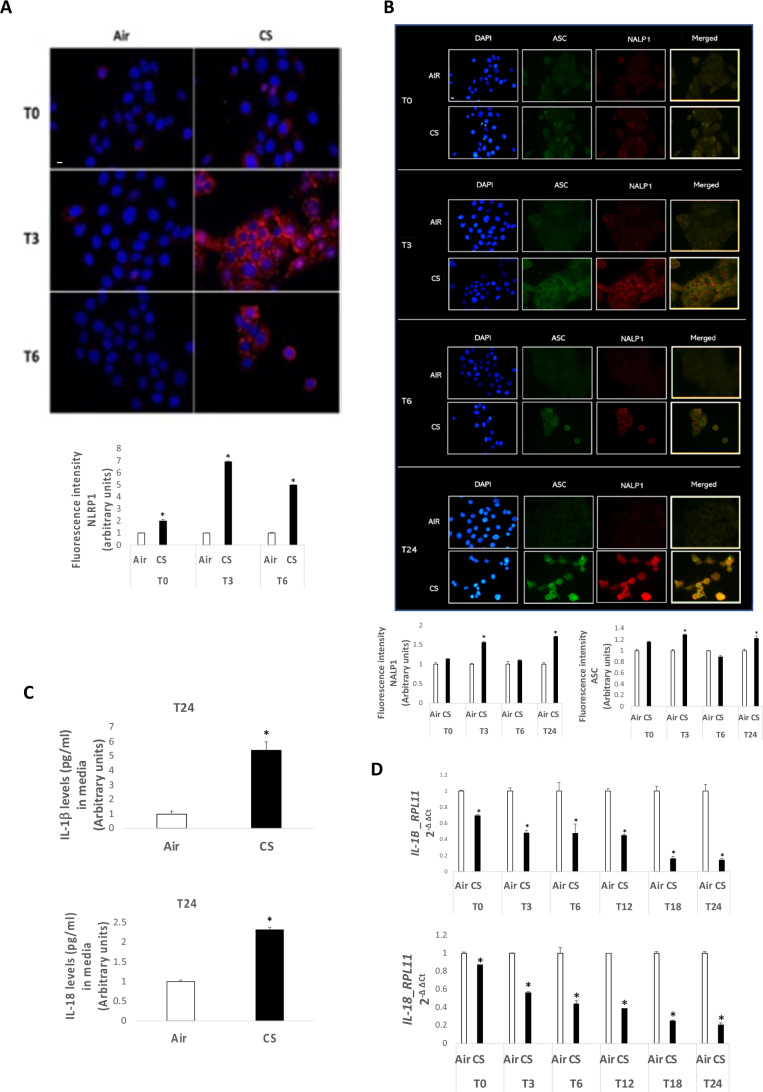
(*A*) Immunocytochemical staining images for NALP1 protein (*red staining*) and nuclei staining (DAPI) in HaCaT cells at 0, 3, and 6 h (T0, T3, and T6) after 15 min of exposure to CS. Images were taken at 40 × magnification,* scale bar* = 20 μm; immunofluorescence signal was quantified using ImageJ software (*bottom panel*). (*B*) Immunocytochemical staining for the co-localization of ASC protein (*green*) and NALP1 (*red staining*) in HaCaT cells 0, 3, 6, and 24 h (T0, T3, T6, and T24) after 15 min of CS exposure or unexposed cells. *Blue staining* (DAPI) represents nuclei; images were taken at 40 × magnification; *scale bar* = 20 μm. The NLRP1-ASC expression levels were measured by using ImageJ software (*bottom panel*). (*C*) Levels of released IL-1β (*upper panel*) and IL-18 (*bottom panel*) (pg/mL) in media of HaCaT cells 24 h post 15 min of CS exposure. (*D*) Transcription levels of *IL-1β* and *IL-18* measured by qRT-PCR in HaCaT samples exposed for 15 min to CS and collected at 0, 3, 6, 12, 18, and 24 h (T0, T3, T6, T12, T18, and T24) upon the end of the 15-min CS exposure. *RPL11* was used as reference gene. Data are the results of the averages of at least three different experiments, *p < 0.05 by one-way ANOVA

Although previous studies have already demonstrated that NLRP3 can be modulated by CS (Pauwels *et al.* 2011; Han *et al.* 2017; Buscetta *et al.* 2020), very little is known about the effect of CS on other types of inflammasomes. Since we found that CS exposure was able to induce higher expression levels of ASC oligomers despite NLRP3 downregulation (Fig. 2*D*), we wonder whether other inflammasomes related to cutaneous tissues could be modulated by CS exposure. NLRP1 is present within the human skin, and it can be modulated by different air pollutants as particulate matter (PM), ozone (O_3_), and UV radiations (Ferrara *et al.* 2021). NLRP1 similarly to NLRP3 can recruit ASC when activated to perpetuate the inflammatory response but it does not require the priming step and the activation of NF-κB. As depicted in Fig. 3*A*, CS induced higher expression levels of NLRP1, especially 3 (T3) and 6 (T6) h after CS exposure (Fig. 3*A*). In addition, as it is shown in Fig. 3*B*, NLRP1 colocalized with ASC after CS exposure, confirming the scaffold formation and the inflammasome activation (Fig. 3*B*). As final step of the inflammasome activation, we measured the levels of IL-1β and IL-18 in keratinocytes exposed to CS. Figure 3*C* shows the increased released levels of IL-1β and IL-18 in the culture media of HaCaT cells after 24 h of CS exposure, while *IL-1β* and *IL-18* gene expression decreased (Fig. 3*D*).

## Discussion

Cigarette smoke is one of the most toxic air pollutants affecting human health, both indoor and outdoor. Besides the respiratory tract, among which lungs are the main targeted tissue, the skin represents another important target for CS insult, due to its location and the continuous exposure to environmental stressors (Woodby *et al.* 2020). Although, the exact mechanism of CS toxicity is not completely established due to its large variety of toxins, CS exposure stands as a potential environmental risk factor for the development or exacerbation of inflammatory skin pathologies. As demonstrated for other outdoor stressors, CS exposure can affect skin homeostasis through the generation of oxidative and inflammatory responses, which can fuel each other causing OxInflammation, and possibly develop inflammatory-related skin conditions (Sticozzi *et al.* 2012a, b; Valacchi *et al.* 2018). Since both inflammation and oxidative stress are displayed in several cutaneous pathologies as well as in skin aging, the role of pollutants in inducing cutaneous damage has been long investigated (Mancebo and Wang 2015; Puri *et al.* 2017; Parrado *et al.* 2019; Hieda *et al.* 2020). In the past decades, inflammasomes have been found to be important immune system sensors to mediate the inflammatory response in several conditions including skin abnormalities and cancer (Dombrowski *et al.* 2011; De Sá and Festa Neto 2016; Awad *et al.* 2018). Inflammasome activation can be triggered by numerous stimuli including air pollutants, such as particulate matter (PM) but also UV radiations, whom detrimental effect has also been associated to the onset of a variety of skin conditions (Ferrara *et al.* 2021). However, the role of inflammasomes in mediating cutaneous damage induced by pollutants is still poorly understood. NLRP3 is one of the most studied inflammasomes, and it can be activated by a variety of stimuli. Its involvement has been now demonstrated in the pathogenesis of numerous inflammatory conditions, including the one related to skin (Heneka *et al.* 2013; Wang *et al.* 2020). Although other air pollutants have been shown to activate NLRP3 inflammasome and mediate pulmonary, cardiovascular, and neurodegenerative diseases (Saresella *et al.* 2016; Zheng *et al.* 2018; Du *et al.* 2019), the effect of CS on the activation/inhibition of NLRP3 is still controversial and under debate. For instance, NLRP3 has been found to be negatively modulated by CS exposure in human THP1 cells and in mice (Han *et al.* 2017; Buscetta *et al.* 2020), whereas other studies indicate NLRP3 activation upon CS insult in bronchial epithelial cells and other pathologies as atherosclerosis, vascular and bladder dysfunctions, or brain injury (Li *et al.* 2019; Mehta *et al.* 2020; Rumora *et al.* 2020; Wu *et al.* 2020; Ma *et al.* 2021). To our knowledge, the effect of CS on assembly and activation of skin inflammasome has never been described yet. Thus, the aim of the present study was to investigate whether the detrimental cutaneous effect of CS could be mediated also by the skin inflammasomes activation. For this purpose, HaCaT cells were chosen as a reliable epithelial model to study keratinocyte responses as they derived from normal human skin and are non‐tumorigenic and able to maintain full differentiation capacity, representing an appropriate model to investigate the responses of chemicals (Phillips *et al.* 2008; Bonifas *et al.* 2010). It is well known that CS contains high levels of ROS (Avezov *et al.* 2014). And our first results confirmed that CS exposure could induce higher levels of 4-HNE (Fig. 1*B*), indicating an increase in cellular oxidative stress upon CS exposure on human skin. Indeed, 4-HNE is a reactive aldehyde produced as result of lipid peroxidation triggered by the interaction of oxidative species induced by pollutants and the lipids present within the cell membrane (Codreanu *et al.* 2009; Sottero *et al.* 2018; Woodby *et al.* 2020). 4-HNE can interact with proteins resulting in post-translational modifications (Muresan *et al.* 2015), which lead to the changing of the protein conformation and thereby the alteration of their function that eventually evolves in aberrant cellular responses (Poli *et al.* 1982; Grune *et al.* 1997; Uchida 2003; Pecorelli *et al.* 2011). In addition, the altered redox status was also confirmed by increased levels of carbonyls, suggesting the ability of CS not only to induce lipid peroxidation but also to oxidize proteins as shown in smokers (Dalle-Donne *et al.* 2017). Since CS exposure has been shown to modulate the activation of redox-sensitive factors related to an inflammatory response, we decided to investigate the possible involvement of the NF-κB in CS-mediated skin inflammatory responses in keratinocytes. NF-κB has been reported to be the common pathway for the conversion of environmental insults into cutaneous inflammation. Perturbations in its activity such as overactivation or inhibition are linked to the development of skin defects, inflammatory skin disease, and skin cancer (Sticozzi *et al.* 2012a, b; Magnani *et al.* 2016; Ferrara *et al.* 2020). NF-κB acts in immune and non-immune cells to control the maintenance of tissue immune homeostasis which is maintained by an extensive cross-talk between epidermal keratinocytes and immune cells (Romani *et al.* 2018). In the basic state, NF-κB exists in the cytosol as a pre-formed trimeric complex, a p50/p65 protein dimer associated with an inhibitory protein IKB. Upon different stimuli, NF-κB transcription factor is phosphorylated, released, and subsequently translocated to the nucleus to induce the transcription of many genes whose proteins are involved in the inflammatory responses to stress. Downstream products of NF-κB activation include inflammatory cytokines such as IL-8, IL-6, IL-1β, and IL-18. Nowadays, there is discrepancy between the published data on the effects of CS on NF-κB activation in the respiratory tract since some findings have highlighted a NF-κB activation, whereas others found inhibition or no effect (Laan *et al.* 2004; Castro *et al.* 2008; Manzel *et al.* 2011). This opposite effect could also be related to the amount of ROS and aldehydes present in CS and produced after interaction with biological matrix. For instance, acrolein, which is a major product of organic combustion such as tobacco smoke and represents the most reactive alpha, beta-unsaturated aldehyde in CS, has been shown to inhibit NF-κB activation by interacting directly with IKKβ subunit and preventing the translocation of p65 to the nucleus (Valacchi *et al.* 2005). Therefore, it is possible that also in our case, the lack of NF-κB translocation detected in keratinocytes could be a consequence of the interaction between the aldehydes produced by CS combustion and IKK. Moreover, NF-κB has been demonstrated to be essential for the activation of certain types of inflammasomes, such as NLRP3. Indeed, NLRP3 activation relies on a two-step mechanism which consists of a priming step involving the activation of NF-κB upon infectious stimuli (i.e., LPS, TNF-α) to induce the transcription of inflammasome components as *NLRP3* itself and interleukins as IL-1β and IL-18. Once transcribed, NLRP3 can be activated by an array of stimuli (i.e., K^+^ efflux, mitochondrial stress, ROS, etc.) eventually leading to the recruitment of the speck-like receptor ASC, the consequent activation of the caspase-1 enzyme, and the maturation of the inflammatory interleukins IL-1β and IL-18 (Hornung and Latz 2010; Lamkanfi and Dixit 2014; Guo *et al.* 2015; Vanaja *et al.* 2015). In our experimental conditions, we demonstrated that CS exposure inhibits not only NLRP3 expressions but, as expected, also the activation of caspase-1 (Fig. 2*C*, *E*). Interesting, although we detected an NLRP3 inhibition, we encountered increased ASC speck oligomers, 3 and 6 h after CS exposure (Fig. 2*D*), suggesting that, possibly, other inflammasomes could be involved in the CS-induced inflammatory response in human skin. Several studies demonstrated the cross-talk between different types of inflammasomes, canonical and non-canonical such as caspase-11 and the human orthologs caspase-5 and caspase-4 (Vanaja *et al.* 2015; Zanoni *et al.* 2016; Yi 2020). For instance, the inhibition of NLRP3 inflammasome can trigger the activation of other pathways as the TLR4-TRIF-caspase-8 axis in human macrophages (Buscetta *et al.* 2020). The inflammasome NLRP1 has been reported to be strongly expressed in keratinocytes, representing a crucial skin immune sensor (Khare *et al.* 2010; Burian and Yazdi 2018). Genetic mutations in NLRP1 have been associated with hyperplasia and skin autoimmune disease like vitiligo accompanied with high levels of IL-1β and IL-18 detected in the serum of patients (Levandowski *et al.* 2013; Marie *et al.* 2014; Zhong *et al.* 2016). Several studies have also demonstrated that pollutants such as ozone, PM, and UV radiations can induce NLRP1 activation in the cutaneous tissue, most likely through oxidative stress reactions (Awad *et al.* 2018; Ferrara *et al.* 2019; Dong *et al.* 2020). Interestingly, we found that CS exposure was able to induce NLRP1 activation in keratinocytes. Furthermore, an evident co-localization of NLRP1 and ASC occurred especially 3, 6, and 24 h upon the CS insult, suggesting that the increased ASC oligomer expression levels found in keratinocytes exposed to CS could be due to the activation of NLRP1. In addition, we found that CS exposure increased released levels of IL-1β and IL-18 in culture media of keratinocytes exposed to CS. This data suggests that the delayed NLRP1 inflammasome activation could represent a compensatory mechanism to cope the initial NLRP3 inflammasome inhibition by CS exposure. Indeed, although CS seemed to inhibit caspase-1 activation, it should be mentioned that NLRP1 inflammasome does rely not only on caspase-1 activation to ensure IL-1β and IL-18 maturation, but also on other types of non-canonical caspases, such as caspase-5, as initially described by Martinon *et al.* (2002). It has been demonstrated that the release of IL-1β and IL-18 in autoinflammatory skin diseases related to NLRP1 inflammasome activation depends on caspase-5 activation rather than caspase-1 (Zwicker *et al.* 2017) and that caspase-4 expression is required for UVB-induced activation of pro-IL-1β and IL-18 in skin-derived keratinocytes (Sollberger *et al.* 2012). Thus, NLRP3 and other inflammasomes might cooperate to respond to the CS exposure in human keratinocytes and ensure an inflammatory response. In addition, the inhibition of NLRP3 upon CS exposure could be due to the ability of pollutants to induce post-translational modifications in their targets, including ubiquitination of altered proteins. Ubiquitination has been found to inhibit the activation of the NLRP3 inflammasome (Han *et al.* 2017), whereas recent studies have demonstrated that ubiquitination of NLRP1 is necessary for its full activity in mice (Mitchell *et al.* 2019; Sandstrom *et al.* 2019; Xu *et al.* 2019). In conclusion, our study suggests that CS inhibits NLRP3 inflammasome in human keratinocytes, which can possibly, directly or indirectly, promote the activation of other inflammasome pathways, canonical and non-canonical, that might cooperate in the context of an inflammatory response within the cutaneous tissue. It is plausible to think that CS could first inhibit NLRP3 inflammasome and subsequently activate NLRP1 via ubiquitination. Future studies are needed to better investigate the activation of the NLRP1-caspase-5 axis as well as other non-canonical pathways in response to CS to better understand the role of this air pollutant in inflammasome activation related to human skin pathologies.
